# Endogenous laminin is required for human airway smooth muscle cell maturation

**DOI:** 10.1186/1465-9921-7-117

**Published:** 2006-09-12

**Authors:** Thai Tran, Karol D McNeill, William T Gerthoffer, Helmut Unruh, Andrew J Halayko

**Affiliations:** 1 Departments of Physiology and Internal Medicine, University of Manitoba, Winnipeg, MB, Canada; 2 Biology of Breathing Group, Manitoba Institute of Child Health, Winnipeg, MB, Canada; 3 CIHR National Training Program in Allergy and Asthma, University of Manitoba, Winnipeg, MB, Canada; 4 Department of Pharmacology, University of Nevada School of Medicine, Reno, NV, USA; 5 Section of Thoracic Surgery, University of Manitoba, Winnipeg, MB, Canada; 6 Section of Respiratory Diseases, University of Manitoba, Winnipeg, Canada

## Abstract

**Background:**

Airway smooth muscle (ASM) contraction underlies acute bronchospasm in asthma. ASM cells can switch between a synthetic-proliferative phenotype and a contractile phenotype. While the effects of extracellular matrix (ECM) components on modulation of ASM cells to a synthetic phenotype have been reported, the role of ECM components on maturation of ASM cells to a contractile phenotype in adult lung is unclear. As both changes in ECM components and accumulation of contractile ASM are features of airway wall remodelling in asthma, we examined the role of the ECM protein, laminin, in the maturation of contractile phenotype in human ASM cells.

**Methods:**

Human ASM cells were made senescence-resistant by stable expression of human telomerase reverse transcriptase. Maturation to a contractile phenotype was induced by 7-day serum deprivation, as assessed by immunoblotting for desmin and calponin. The role of laminin on ASM maturation was investigated by comparing the effects of exogenous laminin coated on culture plates, and of soluble laminin peptide competitors. Endogenous expression of laminin chains during ASM maturation was also measured.

**Results:**

Myocyte binding to endogenously expressed laminin was required for ASM phenotype maturation, as laminin competing peptides (YIGSR or GRGDSP) significantly reduced desmin and calponin protein accumulation that otherwise occurs with prolonged serum deprivation. Coating of plastic cell culture dishes with different purified laminin preparations was not sufficient to further promote accumulation of desmin or calponin during 7-day serum deprivation. Expression of α2, β1 and γ1 laminin chains by ASM cells was specifically up-regulated during myocyte maturation, suggesting a key role for laminin-2 in the development of the contractile phenotype.

**Conclusion:**

While earlier reports suggest exogenously applied laminin slows the spontaneous modulation of ASM to a synthetic phenotype, we show for the first time that endogenously expressed laminin is required for ASM maturation to the contractile phenotype. As endogenously expressed laminin chains α2, β1 and γ1 are uniquely increased during myocyte maturation, these laminin chains may be key in this process. Thus, human ASM maturation appears to involve regulated endogenous expression of a select set of laminin chains that are essential for accumulation of contractile phenotype myocytes.

## Background

Remodelling of the airway wall is a feature of chronic asthma and is characterized by a number of structural changes including, but not limited to, increased mass of contractile airway smooth muscle (ASM) [1], and fibrosis resulting from the accumulation of extracellular matrix proteins (ECM) [2,3]. ASM is a key determinant of airway hyperresponsiveness and remodelling in asthma. Airway myocytes are thought to have capacity to contribute to remodelling due to their ability for graded, and reversible phenotype switching, which confers broad functional capacity [4,5]. At one extreme airway myocytes exist in an immature phenotype that is characterised by a high tendency for proliferation, expression and secretion of ECM proteins, and synthesis of inflammatory mediators in response to a number of environmental cues [4-7]. In contrast, myocytes of a mature phenotype serve a primarily contractile function and are marked by a unique repertoire of cytoskeletal and contractile apparatus proteins; including smooth muscle myosin heavy chain, SM22, desmin and calponin [5,7-9]. Notably, however, there is evidence that contractile smooth muscle cells are capable of expressing ECM components such as glycosaminoglycans [10] and collagen [11], suggesting that ASM cells exist in a functional phenotype that is intermediate to the fully synthetic and contractile state.

Laminins are cross-shaped heterotrimeric glycoproteins of the ECM that contain one copy each of an α-, β- and γ-chain [12,13]. The expression of laminin is tissue dependent and varies at different times during development [14]. In the lung, the most significant changes in the expression pattern of laminin occurs between the pseudoglandular and canalicular stage, during which differentiation of ASM cells is initiated and the structural ordering of the airway wall is established [15].

Using antibodies that block laminin polymerisation or receptor binding to laminin, Schuger and colleagues [16,17] showed that lung mesenchymal cell spreading on laminin-containing ECM is required for differentiation of embryonic lung mesenchymal cells into ASM cells. Moreover, similar studies with embryonic mouse organotypic and whole lung cultures reveal laminin is an essential basement membrane component necessary for both pulmonary branching morphogenesis, and for the circumferential alignment of ASM cells around the airway epithelia. Laminin required for ASM differentiation and structural organization of the airway is synthesized, in part, by the developing myocytes themselves, as suppression of α1 laminin chain secretion using brefeldin A prevents myocyte accumulation in the vicinity of developing epithelia [18]. Notably, the airways of asthmatics show greater immunoreactivity for the α2 and β2 laminin chains compared with the airway wall of healthy controls [19]. Also, cultured human ASM cells obtained from asthmatic patients exhibit an altered expression profile of ECM proteins that includes increased collagen type I and decreased laminin α1 chain [20]. Furthermore, cultured human ASM cells stimulated with asthmatic serum produce increased amounts of the γ1 chain of laminin [21]. Collectively, these observations suggest that changes in the endogenous expression of laminin by ASM cells may occur in asthma, and this could be an essential determinant of myocyte phenotype and function during disease pathogenesis.

Hirst and colleagues [22] showed that primary cultured human ASM cells grown on a laminin matrix proliferated much more slowly, were less responsive to mitogens, and expressed greater abundance of contractile proteins compared with cells grown on a plastic or collagen type I matrix. This suggests that exogenous laminin prevents the spontaneous modulation of contractile ASM cells to a synthetic/proliferative phenotype in cell culture. Given the emerging evidence for ASM phenotype plasticity and its potential association with pathogenesis of features of asthma such as the accumulation of contractile ASM mass and fibrosis of the airway wall, in the current study we investigated whether laminin was required for the maturation of ASM cells to a contractile phenotype in culture. For our studies we used prolonged serum deprivation to induce contractile phenotype maturation in human ASM as we have previously described [9,23-25]. Based on expression of desmin and calponin, which are well characterised protein markers for the mature/contractile phenotype [8,26-29], we tested the requirement of endogenously expressed laminin for contractile myocyte maturation. By elucidating the cellular and molecular mechanisms that regulate airway myocyte differentiation and phenotype modulation we hope to better understand the role of laminin in the contribution of ASM cells to the pathogenesis of asthma.

## Methods

### Immortalized human airway smooth muscle cell culture

For all studies at least four senescence-resistant human airway smooth muscle (HASM) cell lines were prepared using MMLV retroviral vectors to facilitate stable integration of the human telomerase reverse transcriptase gene (hTERT) as we have previously described [25]. The primary cultured HASM cells used to generate each cell line were prepared as we have previously described from macroscopically healthy segments of 2nd-to-4th generation main bronchus obtained after lung resection surgery from patients with a diagnosis of adenocarcinoma [30,31]. All procedures were approved by the Human Research Ethics Board (University of Manitoba). hTERT-expressing HASM cells retain the ability to express markers of the contractile phenotype including smooth muscle myosin heavy chain, calponin, sm-α-actin, and desmin to passage 10 and higher [25]. For all experiments, passages 16–22 cultures were used. To induce 'contractile/mature' phenotype expression in subpopulations of hTERT-HASM, confluent cultures were maintained in Dulbecco's Modified Eagle's Medium (DMEM) supplemented with ITS (insulin 5 μg/ml; transferrin 5 μg/ml, selenium 5 ng/ml) for up to 7 days as we have described [9].

### Experimental system

Human ASM cells were seeded onto 100 mm dishes and left to grow to monolayer confluence in DMEM containing 10% v/v foetal bovine serum (FBS). The cells were then trypsinised and replated onto 6-well plastic, or laminin-coated culture dishes at a seeding density of 1 × 10^5 ^cells per well in DMEM containing 0.5% v/v FBS (preliminary experiments confirmed that this seeding density was sufficient to retain confluency in re-seeded cultures). Before subsequent treatments, re-plated cultures were incubated at 37°C for ~16 hrs, to ensure full cell adherence and spreading [22,32] independent of any influences on ASM proliferation induced by the ECM (as measured from cell counts using the haemocytometer). To induce a contractile phenotype, cells were then serum deprived for up to 7 days in DMEM/ITS, a duration previously shown by our laboratory to be sufficient to induce myocyte maturation and increased expression of the smooth muscle contractile apparatus protein; calponin, and the intermediate filament protein, desmin [9,33,34]. Serum-free media was replaced with fresh DMEM/ITS every second day.

To examine the role of laminin in the maturation of HASM cells, laminin-competing peptides (1 μM) were added at the time of serum deprivation and re-added every second day when fresh serum-free media was replaced. The YIGSR pentapeptide corresponds to the 929–933 sequence of the β chain of laminin [35] and is found to compete with laminin for binding to the laminin receptor. The YIGSR peptide (Sigma, Saint Louis, MO) was reconstituted in distilled water to a stock concentration of 10 mM and then diluted to 1 μM final concentration in serum-free DMEM for use in experiments. The GRGDSP peptide is derived from the amino acid sequence of the α chain of laminin. The RGD amino acid sequence is also found within fibronectin, where it was originally identified as the sequence motif that mediates cell attachment [36]. The GRGDSP and GRADSP peptides (Calbiochem, La Jolla, CA) were reconstituted in 5% acetic acid to a stock concentration of 10 mM and then for use in experiments was diluted in serum-free DMEM to 1 μM final concentration as per previous reports [37]. In pilot experiments (not shown), we determined that the highest concentration of vehicle acetic acid (0.0005%) was well below the threshold concentration for influencing HASM cell proliferation, desmin or calponin protein accumulation.

### Laminin coating of culture plates

To examine the effect of exogenous laminin, plastic cell culture plates were coated with various forms of laminin according to previously described methods [22]. For coating experiments, all laminin preparations were reconstituted in sterile PBS and then diluted to 10 μg/ml in PBS. Briefly, each laminin preparation was adsorbed to 6-well plates for 6 hrs at room temperature. Non-specific binding sites were blocked for 30 minutes with PBS containing 0.1% w/v BSA, at room temperature. Prior to seeding of cells onto the laminin-coated plates, the plates were washed with DMEM. The laminin preparations used included laminin prepared from Engelbreth-Holm-Swarm (EHS) murine sarcoma (Sigma, St. Louis, MO) that predominately consists of the laminin trimer α1, β1 and γ1. The degree of homology between full length murine and human laminin is at least 78%, with laminin receptor binding domains exhibiting 100% homology [14,38]. Two laminin preparations from freshly frozen human placenta tissue were also used (gift from Dr J. Wilkins of the University of Manitoba, Canada). Placental laminin was isolated by affinity purification with monoclonal 4E10 or 5H2 antibodies, which recognize laminin β1 and α2 chains, respectively [39,40].

### Measurement of ASM mature-marker proteins

For analysis of myocyte phenotype, protein lysates were harvested 16 hrs after re-plating (basal/Day 0) and following 7 days serum-deprivation (Day 7). Cells were washed twice with ice-cold PBS and extracted with ice-cold lysis buffer (100 mM NaCl; 10 mM Tris-HCl, pH 7.5; 2 mM EDTA; 0.5% w/v deoxycholate; 1% v/v triton X-100; 1 mM phenylmethylsulphonylfluoride; 10 mM MgCl_2_; 5 μg/ml aprotinin; 100 μM sodium orthovanadate). The cells were scraped, transferred to 1.5 ml plastic tubes, centrifuged (760 × g, 5 min) and the supernatant stored at -20°C. Protein content in supernatant samples was determined using the Bio-Rad protein assay (BioRad, Hercules, CA). The samples (12–15 μg protein per lane) were separated electrophoretically under reducing conditions on an 8% SDS-polyacrylamide gel and proteins were transferred onto nitrocellulose membranes for western blotting. Membranes were blocked with 5% w/v skim milk in Tris Buffered Saline (10 mM Tris HCl, pH8, 150 mM NaCl) and 0.1% v/v Tween-20, then incubated with primary antibodies, diluted in 1% w/v skim milk, to desmin (1:500 dilution) or calponin (1:2,000 dilution). The membranes were developed by subsequent incubation with secondary horseradish-peroxidase-conjugated antibody, then visualized with enhanced chemiluminescence reagents (Amersham, Buckinghamshire, UK). Membranes were re-probed with antibodies for β-actin to normalize for equal loading of all samples. Scanning and quantification of the relative protein abundance was performed using the Epson Perfection 4180 Station and TotalLab TL100 software (Nonlinear Dynamics, Durham, NC). Protein results were expressed as fold increment over basal (Day 0) relative to β-actin.

### Real-Time PCR

Total RNA was extracted from hTERT-HASM cells cultured in 6-well plates using the Qiagen RNeasy Mini Kit (Qiagen, Mississauga, ON) according to the manufacturer's protocol. Total RNA (2 μg) was reversed transcribed using M-MLV reverse transcriptase (Promega, Madison, WI), incubated for 2 h at 37°C followed by 5 min incubation at 95°C, and diluted 1:10 with RNase-free water. The cDNA sample was further processed by Real-Time PCR using the primer pairs listed in Table 1. Cycle parameters were: denaturation at 92°C for 45 s, annealing at 60°C for 45 s and extension at 72°C for 90 s for 40 cycles. Assays were performed in duplicate in 20 μl reactions and the cycle threshold (C_T _= amplification cycle number) values for each reaction were determined using Roche Molecular Biochemicals LightCyler 3 (version 3.5). Real-Time PCR data was analysed using the comparative C_T _method as previously described [41]. The amount of target gene normalised to an endogenous reference (18s rRNA, designated as ΔC_T_) and relative to a calibrator (Day 0, designated as ΔΔC_T_) is given by the equation 2^-ΔΔCT^.

**Table 1:** List of primers for laminin chains used in Real-Time PCR

***Gene product***	***NCBI accession number***	***Primer sequences***
Laminin α1 chain	NM005559	Forward 5'TGG GTG TGG GAT TTC TTA GC 3' Reverse 5'CCT GAC CGT CTA CCC AGT GT 3'
Laminin α2 chain	NM000426	Forward 5'GGC TTA TTC AGC TGG CAG AG 3' Reverse 5'ATT CTC CCA GGG ACT TTG CT 3'
Laminin β1 chain	NM002291	Forward 5'AAC GTG GTT GGA AGA ACC TG 3' Reverse 5'ACA CTC CCT GGA AAC AGT GG 3'
Laminin β2 chain	NM002292	Forward 5'CCT AGC CCT GTG AGC AAC TC 3' Reverse 5'GTC TGT CAG GCT CAG GGT GT 3'
Laminin γ1 chain	NM002293	Forward 5'AAT CCG TAT GGG ACC ATG AA 3' Reverse 5'TCA CAC CTC TCA CAG CCT TG 3'
18s rRNA	[41, 89]	Forward 5'CGC CGC TAG AGG TGA AAT TC 3' Reverse 5'TTG GCA AAT GCT TTC GCT C 3'

### Materials

All chemicals used were of analytical grade or higher. All compounds [mouse anti-calponin antibody (clone hCP, C2687), YIGSR, laminin-EHS] were purchased from Sigma (St. Luois, MO) unless stated otherwise. GRGDSP and GRADSP peptides were obtained from Calbiochem (EMB Biosciences Inc. La Jolla, CA). Rabbit anti-desmin antibody (H-76, sc14026) was purchased from Santa Cruz Biotechnology Inc (Santa Cruz, CA). Antibodies to laminin chains α2, β1, β2 and γ1 were a gift from Dr. Eva Engvall (The Burnham Institute, La Jolla, CA, USA).

### Statistical analysis

Data are expressed as mean and standard error of the mean (SEM) of observations obtained from ASM cells cultured from at least four different cell lines. All experiments were carried out in duplicate. Data were expressed as fold increment over basal (Day 0) relative to β-actin (protein) or 18s rRNA (mRNA) and results were analysed using one-way ANOVA, with repeated measures, followed by Bonferroni's post hoc t test, where appropriate. A probability value of *P *< 0.05 was considered significant.

## Results

### Effect of blocking laminin binding on HASM maturation

**Figure 1:**
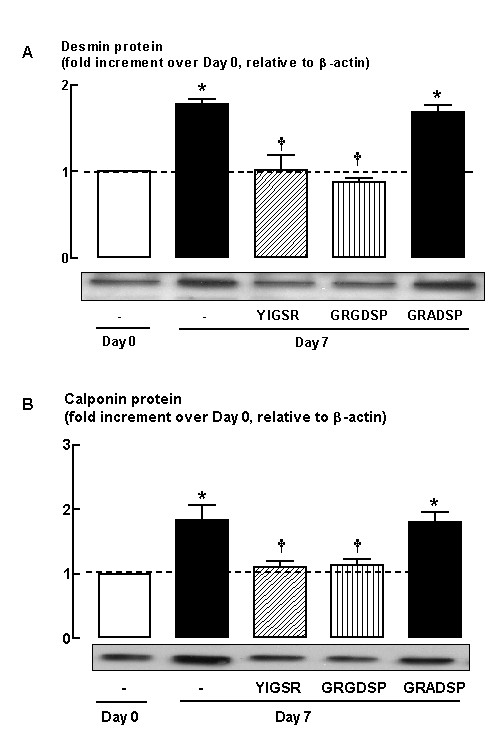
Effect of laminin-competing peptides (YIGSR, 1 μM and GRGDSP, 1 μM) on (A) desmin and (B) calponin protein abundance following 7-day serum deprivation. YIGSR = peptide derived from the amino acid sequence of the β1 chain of the major receptor binding site in laminin; GRGDSP = amino acid sequence within fibronectin and other extracellular proteins that mediates cell attachment; GRADSP = inactive peptide for GRGDSP. Grouped data represent results obtained from three different cultures. * *P *< 0.05, compared with Day 0; † *P *< 0.05 compared with Day 7 response in the absence of peptide.

We first assessed phenotype maturation by measuring the abundance of stringent contractile phenotype marker proteins desmin and calponin [5,8]. Under basal conditions, 16 hrs after replating myocytes from confluent cultures in DMEM containing 0.5% FBS, HASM cells expressed low levels of desmin and calponin. However, following 7-days in serum deficient conditions, both desmin and calponin protein increased markedly, exhibiting a doubling in abundance (*P *< 0.05, Figure 1). This expression pattern is consistent with phenotype maturation of ASM cell myocytes that we have described previously for both hTERT immortalized cells and primary cultured airway smooth muscle cells [5,8,25].

**Figure 2:**
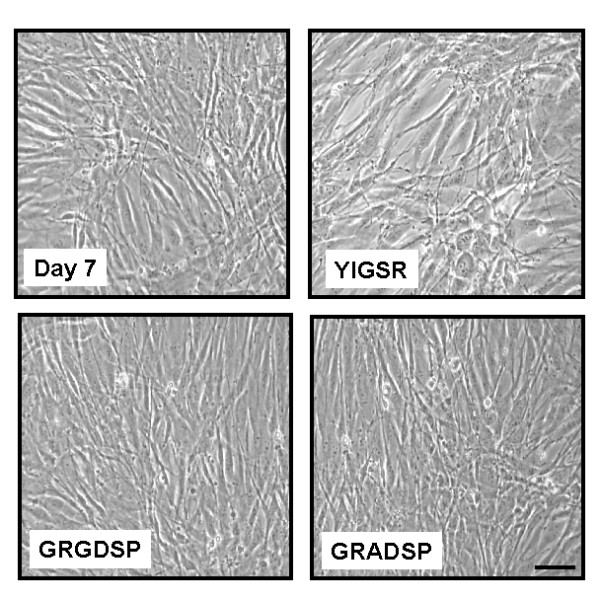
Phase contrast images of ASM cells in the presence and absence of laminin-competing peptides (YIGSR, GRGDSP, GRADSP, 1 μM) following 7-day serum deprivation. YIGSR = peptide derived from the amino acid sequence of the β1 chain of the major receptor binding site in laminin; GRGDSP = amino acid sequence within fibronectin and other extracellular proteins that mediates cell attachment and also correspond to the α chain of laminin; GRADSP = inactive peptide for GRGDSP. Bar = 70 μm.

We first examined whether laminin was required for HASM maturation. HASM cells were incubated with laminin mimetic peptides (1 μM), YIGSR and GRGDSP, that competitively inhibit binding of endogenously expressed laminin. The peptides were added ~16 hrs after replating myocytes from confluent cultures and at the same time that 0.5% FBS-supplemented DMEM was replaced with serum-deficient DMEM; thereafter peptides and culture media was replaced every second day. The peptide YIGSR is a selective inhibitor of laminin as it corresponds to a unique amino acid sequence on the laminin β1 chain [42]. YIGSR has previously been reported to promote cell attachment and migration, and to block angiogenesis and tumour metastases [42]. The GRGDSP peptide is semi-selective for blocking laminin binding, as it corresponds to the amino acid sequence on the short arm of the α chain of laminin, which has sequence homology with the binding region of other ECM proteins such as fibronectin [43]. Selective blockade of laminin binding with YIGSR (1 μM) abrogated the accumulation of desmin and calponin protein abundance that occurred in untreated cultures after 7 days serum deprivation (Figure 1). Similarly, incubation of cultures with GRGDSP prevented the accumulation of the contractile phenotype marker proteins desmin and calponin (Figure 1). Conversely the negative control peptide, GRADSP [36] had no effect on phenotype maturation, as desmin and calponin protein accumulation following 7-day serum deprivation was similar to untreated controls (Figure 1). Importantly, the inhibitory effect of YIGSR and GRGDSP was not the result of peptide-induced cell toxicity as trypan blue exclusion was not increased compared to untreated cultures. Neither YIGSR nor GRGDSP induced detectable cell detachment or cell rounding, and had little effect on myocyte morphology, as seen by phase contrast imaging (Figure 2). Moreover, cell number was unchanged between all treatment groups (untreated cells at Day 7 = 1.0 ± 0.1 × 10^5^; treated cells: YIGSR = 1.0 ± 0.3 × 10^5^; GRDGSP = 1.0 ± 0.3 × 10^5^; GRADSP = 1.0 ± 0.2 × 10^5 ^cells per well). Collectively, these experiments reveal endogenously expressed laminin is required for phenotype maturation of HASM cultured in serum-free conditions.

### Effect of exogenous laminin on HASM maturation

**Figure 3:**
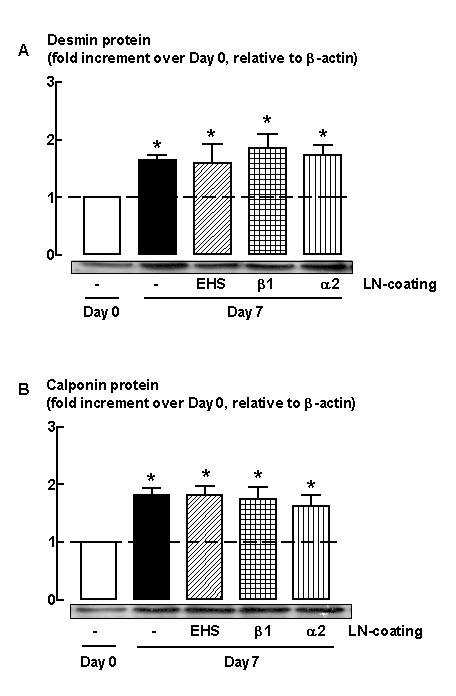
Western blot analysis showing the effect of various types of laminin (LN) on (A) desmin and (B) calponin protein abundance. HASM cells were seeded onto plastic or laminin (10 μg/ml)-coated dishes and then serum deprived for 7 days. EHS = laminin from Engelbreth-Holm-Swarm murine sarcoma; β1 = affinity purified β1 chain-laminin from human placenta that includes LN-1, 2, 6, 8, 10; α2 = affinity purified α2 chain-containing laminin from human placenta that includes LN-2 and 4. Grouped data represent results obtained from at least four different cultures. *P < 0.05, compared with Day 0 on plastic.

As previous work [22] clearly demonstrates that coating of cell culture plates with exogenous laminin inhibits modulation of HASM to a proliferative phenotype, we next examined whether coating of culture dishes with laminin was sufficient to promote phenotype maturation and increase the accumulation of desmin and calponin that occurs with prolonged culture in serum-free conditions. HASM cells were thus re-plated from serum-fed confluent cultures on dishes pre-coated with different laminin preparations, including (1) laminin from EHS tumours that contains laminin type 1 (laminin 111) isoform, (2) affinity purified β1 chain-containing laminin from human placenta that includes laminin-1 (laminin 111), 2 (laminin 211), 6 (laminin 311), 8 (laminin 411), 10 (laminin 511) isoforms, and (3) affinity purified α2 chain-containing laminin from human placenta that consists of laminin-2 (laminin 211) and 4 (laminin 221). Cells were maintained for 7 days in serum deficient media and the accumulation of desmin and calponin was then measured by immunoblotting. Coating culture dishes with exogenous laminin had no effect on HASM cell maturation, as the change in abundance of desmin and calponin was not different from that measured for control cultures in which cells were plated directly onto uncoated plastic culture dishes (Figure 3).

**Figure 4:**
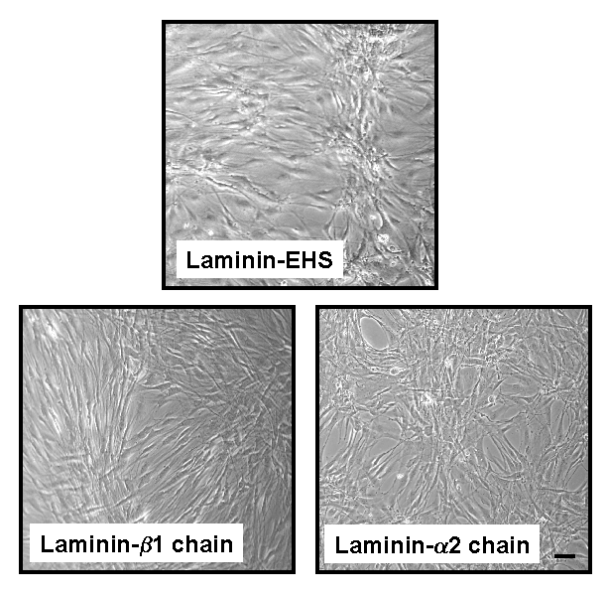
Phase contrast images of ASM cells in the presence and absence of different coating of laminin isoforms following 7-day serum deprivation. EHS = laminin from Engelbreth-Holm-Swarm murine sarcoma; β1 = affinity purified β1 chain-containing laminin from human placenta that includes LN-1, 2, 6, 8, 10; α2 = affinity purified α2 chain-containing laminin from human placenta that includes LN-2 and 4. Bar = 70 μm.

For our studies we used a coating concentration of laminin that was based on pilot experiments in which we constructed a laminin-EHS concentration response curve (0.01–10 μg/ml) that optimised HASM attachment and adherence in DMEM/0.5% FBS culture media. The concentration that we used for our subsequent studies (10 μg/ml) was comparable to that used previously by other groups [22,44]. Notably, in our studies the cell density following 7-day serum deprivation was not different between HASM cells seeded onto plastic or laminin (untreated cells = 1.0 ± 0.1 × 10^5 ^cells compared with cells seeded onto laminin-EHS = 1.0 ± 0.4 × 10^5 ^cells per well). Furthermore, coating with the different laminin preparations that we employed appeared to be equally effective, as for all preparation HASM cells became organized into a web-like pattern that is characteristic of smooth muscle grown onto laminin coated dishes [22,44] (Figure 4). Collectively, our experiments demonstrate that exogenously applied laminin is not sufficient to promote HASM maturation that occurs during prolonged growth in serum-deficient culture conditions.

### Profile of endogenous laminin chains synthesized by HASM cells

**Figure 5:**
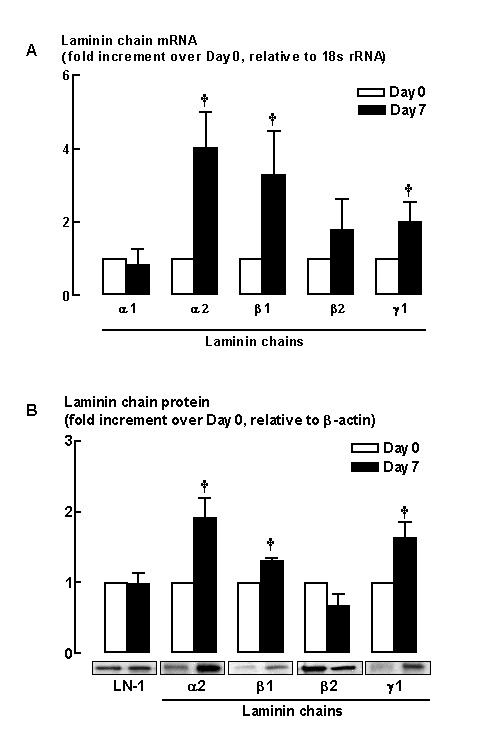
(A) mRNA expression of laminin chains by HASM cells at Day 0 (open bars) and following 7-day serum deprivation (closed bars). Grouped data represent results obtained from three different cultures carried out in duplicate. (B) Western blot analysis showing the protein abundance of laminin chains at Day 0 (open bars) and following 7-day serum deprivation (closed bars). † *P *< 0.05, compared with the respective laminin chain at Day 0.

As our studies with laminin-binding peptide inhibitors (Figure 1) indicate endogenously expressed laminin is required for HASM maturation to a contractile phenotype, we next assessed the pattern of laminin chains that are expressed during 7-days serum deprivation. Real-Time PCR analysis of mRNA from myocytes under Day 0 basal conditions (16 hrs after re-plating from confluent serum-fed cultures) revealed that both α1 and α2 laminin chains are expressed, with the latter being the more abundant (Table 2). In addition, abundant levels of mRNA for β1, β2 and γ1 laminin chains of approximately equal magnitude were expressed by HASM cells (Table 2). Interestingly, following 7-days serum deprivation, the level of expression of the α2, β1 and γ1 laminin chain mRNAs was increased by 4-, 3- and 2-fold, respectively, compared to basal levels at Day 0 prior to serum deprivation (Figure 5A). An increase in the abundance of mRNA for α2, β1 and γ1 laminin chains was observed by 3-days serum deprivation (data not shown) but this was much less marked than at day 7 when our studies were completed. In contrast, abundance of mRNA for α1, β2 laminin chains was unchanged following serum deprivation (Figure 5A). We also performed complementary immunoblotting analyses to assess whether changes in mRNA were reflected in the abundance of the protein encoded by individual laminin chain transcripts. Indeed the trend for increased expression of both α2 and β1 laminin chains that we observed at the mRNA level was mirrored at protein level where α2 abundance was doubled and β1 was increased 30% (*P *< 0.05, Figure 5B). Furthermore, protein for γ1 laminin chain was also increased by 55% (*P *< 0.05).

**Table 2:** Absolute ΔC_T _values for laminin chain mRNA expression

***Gene product***	Laminin chain mRNA (absolute ΔC_T _values)	
	
	***Day 0***	***Day 7***
Laminin α1 chain	22.2 ± 0.5	23.5 ± 0.9
Laminin α2 chain	10.8 ± 0.9	8.8 ± 0.4
Laminin β1 chain	9.0 ± 0.5	7.5 ± 0.7
Laminin β2 chain	9.3 ± 0.3	8.8 ± 0.9
Laminin γ1 chain	8.8 ± 0.4	7.9 ± 0.6

C_T _= threshold cycle/amplification cycle number. ΔC_T _= difference between C_T _value for laminin chain gene of interest versus matched 18s rRNA C_T _value. The smaller the ΔC_T _value the greater the mRNA expression. These absolute ΔC_T _values were used to calculate fold differences in mRNA levels presented in Figure 5 using the comparative C_T _method.

## Discussion

This study was completed to extend understanding of the role of laminin in phenotype expression of human ASM cells from the adult lung. There is ample evidence that laminin has both anti-proliferative effects and slows spontaneous phenotype *modulation *of airway, visceral and vascular smooth muscle cells [22,45-47]. However, despite a number of elegant studies investigating mesenchyme differentiation in airways of embryonic mouse lung cultures [18], the direct role of laminin in phenotype *maturation *of differentiated airway myocytes from the mature lung has not been dissected. Our current studies using soluble peptide inhibitors of laminin binding sites demonstrate for the first time that laminin chains endogenously expressed by HASM cells are required for maturation to a contractile phenotype. This is supported by our observations that each of the laminin competing peptides used, YIGSR and GRGDSP, completely inhibited accumulation of contractile phenotype protein markers (desmin and calponin), in a well established serum-free cell culture system that promotes myocyte maturation [9,23,25]. Furthermore, we have characterized the profile of laminin chains expressed by our human airway myocyte cultures, and documented that expression of the constituents of laminin-2 (α2, β1, and γ1 chains) increases concomitantly with myocyte maturation. In addition, we show that in contrast to the potential for exogenous laminin coated onto culture dishes to prevent phenotype modulation, it is not sufficient to augment endogenous laminin-dependent maturation of cultured HASM. Collectively, these findings demonstrate an essential role for endogenous laminin in determining phenotype expression of HASM cells from the adult lung, and thus could represent a mechanism for intrinsic regulation of the contribution of HASM to changes in airway structure in health and disease.

Laminin is a trimer consisting of three polypeptide chains, α, β and γ, which possess a number of potential binding sites for receptors including the integrins [48-55] and other non-integrin subtypes [56-58]. A number of studies have used soluble peptides that compete with known sequences for laminin receptors, and these have generated considerable understanding of the role of laminins in cell biology. For our studies we use two active peptides, YIGSR and GRGDSP, and an inactive control, GRADSP to modulate myocyte interactions with the ECM. As in previous studies using other cell systems, the peptide inhibitors for laminin binding that we used were well suited for *in vitro *investigation, and thus provided an important tool to determine the role of endogenously expressed laminins in HASM phenotype maturation. None of the peptides induced cell death or detachment. Consistent with published studies [32,36,59-61] our preliminary experiments did reveal that both active peptides prevent adhesion of newly plated cells; this observation was a key element for our experimental design, in which we only added peptides in serum free media after re-plated myocytes had fully attached and spread in the presence of 0.5% FBS.

The YIGSR peptide corresponds to amino acids 929–933 of the β chain of laminin [35] and has been used in numerous experimental conditions *in vitro *and *in vivo *[42,62-66]. For example, YIGSR inhibits tumor growth and tumor cell deposition in the bone, liver, and kidney in a mouse model of B-cell lymphoma [66]. Also, YIGSR reduces the formation of lung colonies in mice injected with melanoma cells and inhibits melanoma cell migration *in vitro *[42]. We used YIGSR as a laminin-specific inhibitor due to its selectively for a unique sequence in the laminin β1 chain. As laminin β1 chain is a component of a number of laminin isoforms we cannot be entirely certain which isoforms were most affected in our study, however based on our expression studies that show evidence for concomitant increase in laminin α2 chain with serum deprivation, it appears that laminin-2 was likely a principal target.

The GRGDSP peptide mimics sequence present in the α chain of laminin that shares homology with a number of other ECM proteins, such as fibronectin [36,61]. Hayman and colleagues [37] showed that the threshold concentration of GRGDSP that induces normal rat kidney cell detachment from fibronectin and vitronectin occurs at concentrations above 1 μM. Though, we used a concentration of 1 μM for all peptides in the current study, the effects of GRGDSP, which were similar to that for YIGSR, cannot be exclusively attributed to the inhibition of laminin binding. Nonetheless, in light of the similarity of the cell responses to YIGSR or GRGDSP in our studies, it is likely that effects of the latter on laminin interactions is a significant element of the responses measured.

Different splice variants of the each of the α, β and γ laminin chains can combine to produce at least 15 functional laminin isoforms. Using immunohistochemical approaches, various laminin chains have been shown to be expressed in the lung and specifically in the airway around smooth muscle bundles [15,19,67-72]. However no studies to date have conclusively shown the presence of distinct staining for specific laminin isoforms due to lack of available antibodies that detect the presence of laminin trimers. We used three different laminin preparations to coat culture dishes and assess the capacity of exogenous laminin to promote phenotype maturation of HASM. EHS laminin is widely used and consists primarily of laminin-1 [12]. As a broader spectrum of laminin isoforms is expressed in the lungs we also used laminin affinity purified from human placenta using β1 laminin chain-selective antibodies. Many of the known laminin isoforms possess a β1 chain, including laminin-1, 2, 6, 8, and 10 [14,69,73]. Laminins-2 (α2 β1γ1), 4 (α2 β2γ1), 8 (α4 β1γ1) and 10 (α5 β1γ1) are expressed in human placenta [39,74,75], thus there is considerable overlap with laminins expressed in the lung. To assess the effects of a more defined lung-relevant laminin we also used an anti-α2 laminin chain affinity purified preparation from human placenta that contained only laminins-2 and 4. Despite using distinct laminin preparations, we did not observe any augmentation of myocyte maturation; the accumulation of desmin and calponin was similar to control cultures. Each laminin preparation induced a web-like HASM cells pattern, which is characteristic of that induced by coating with laminin-1 [22,44]. Collectively, although our studies with competing peptides indicate endogenous laminin is required for HASM maturation, it appears that addition of exogenous laminin is not sufficient to promote this further.

As our studies with competing peptides established a role for endogenous laminin expression in determining HASM phenotype maturation, we profiled laminin chain expression in cultured myocytes. Under serum-fed conditions, characterised by the presence of HASM cells of the proliferative/synthetic phenotype, the profile of laminin chain mRNA consisted predominately of equal abundance of β1, β2 and γ1 laminin chains, whereas α2 and α1 laminin chains were less abundant. This is not unexpected given that the β and γ laminin chains are ubiquitously expressed [69] and α laminin chain expression chiefly contributes to the heterogeneity seen for tissue-specific and developmental stage-specific expression of the laminin isoforms [69,76-79].

Our studies revealed that during HASM maturation, there is a concomitant and significant increase in mRNA and protein for α2, β1 and γ1 laminin chains. These results complement our experiments using α and β1 laminin chain competing peptides that show selective endogenous laminin expression is a determinant of phenotype maturation. That we observed an increased expression of α2, β1, and γ1 laminin with phenotype maturation suggests the involvement of laminin-2 in this process. A number of studies also show that laminin-1 is prominent in the developing lung where it determines mesenchymal cell differentiation, and thereafter laminin-2 accumulates in the adult lung airways [15-17,80,81]. Moreover, our focus on laminin chains that comprise laminin-1 and 2 was due to a lack of available antibodies to perform a comprehensive survey of all α, β and γ laminin chains [69]. Our studies provide new insight concerning endogenous laminin expression by adult HASM cells, and how expression of specific laminin chains, such as α2, β1, and γ1, correlates with changes in HASM phenotype. Moreover, these data suggest that the process of HASM cell maturation may be regulated intrinsically through changes in the synthesis of specific laminin chains.

A large number of studies show that dynamic changes in laminin expression is a key factor in normal lung development [16,80,81], and also in a number of pathologies, including asthma [19,68,82]). In atopic asthmatics whose epithelial integrity is compromised, increased immunoreactivity for γ2 is seen [67]. There are also a number of studies that describe differences in laminin chain composition between allergic asthmatics and non-allergic asthmatics, including evidence for increases in both α2 and β2 laminin chains [19,67,68,83]. Furthermore, Johnson and colleagues [20] report that primary cultured HASM cells exhibit an altered profile of ECM protein expression, including a decrease in laminin α1 chain, that may act as an intrinsic mechanism to promote myocyte proliferation. Similarly, HASM passively sensitized with serum from atopic asthmatics produce increased amounts of the γ1 chain of laminin [21]. In our current study we observed changes in expression of laminin associated with HASM maturation in non-proliferating myocytes. We have previously shown that phenotype maturation is associated with myocyte hypertrophy [84]. Of note, to date there are limited numbers of studies that directly demonstrate ASM cells proliferation *in situ *[85,86], suggesting myocyte hypertrophy contributes significantly to increased muscle mass in airway remodelling. In light of our current study, it is thus tempting to speculate that documented accumulation of ECM around airway smooth muscle [87,88], and changes in laminin chain expression in the airways of asthmatics could contribute to HASM hypertrophy associated with key features of airway remodelling in chronic asthma.

## Conclusion

There are three major new findings from this study: (1) endogenously expressed laminin is both required and sufficient for the maturation of HASM to a contractile phenotype; (2) exogenous laminin (types 1, 2, 3, 4, 6, 8, and 10) are not sufficient to augment the phenotype maturation and accumulation of calponin and desmin that occurs during prolonged serum deprivation of cultured HASM cells; and, (3) HASM cells in culture express a unique profile of laminin chains, and appear to selectively increase expression of α2, β1, and γ1 chains, which comprise laminin-2, in conditions that promote maturation to a contractile phenotype. These results provide strong support for the role of laminin in the maintenance and regulation of ASM phenotype in the adult lung and thus, may be an important mechanism regulating the contribution of myocytes to airways remodelling in disease states such as asthma.

## Competing interests

The author(s) declare that they have no competing interests.

## Authors' contributions

TT carried out the development, implementation and completion of all the experiments as well as the drafting of the manuscript. KDM carried out the set up of primary HASM cells and was involved in the design of PCR primers. WTG prepared the hTERT cell lines. HH organized the provision of lung specimens. AJH is the principal investigator of the study. All authors read and approved the final manuscript.
